# Erythrocyte membrane-encapsulated SZF nanocomposites for hyperuricemia therapy

**DOI:** 10.3389/fimmu.2025.1695253

**Published:** 2026-01-21

**Authors:** Weiwei Liu, Xin Guan, Luobing Wang, Chengjie Zhang, Boyi Shen, Yuke Zeng, Dongdong Li, Guangjin Pu, Jing Hu, Jiandong Gao

**Affiliations:** 1 Department of Nephrology, Shuguang Hospital Affiliated to Shanghai University of Traditional Chinese Medicine, Shanghai, China; 2 Department of Nephrology, Seventh People’s Hospital of Shanghai, University of Traditional Chinese Medicine, Shanghai, China; 3 Traditional Chinese Medicine (TCM) Institute of Kidney Disease, Shanghai University of Traditional Chinese Medicine, Shanghai, China; 4 Key Laboratory of Liver and Kidney Diseases (Shanghai University of Traditional Chinese Medicine), Ministry of Education, Shanghai, China; 5 Shanghai Key Laboratory of Traditional Chinese Clinical Medicine (20DZ2272200), Shanghai, China; 6 Preventive Treatment Department, Xiangshan Traditional Chinese Medicine Hospital, Shanghai, China; 7 Department of Nephrology, The Second Affiliated Hospital of Henan University of Chinese Medicine, Zhengzhou, China; 8 Department of Vascular Surgery, Shanghai Fourth People’s Hospital Affiliated to Tongji University, Shanghai, China

**Keywords:** hyperuricemia, PDA, ROS scavenging, SHP2/ANT1 signaling pathway, SZF

## Abstract

**Introduction:**

Hyperuricemia is a prevalent metabolic disorder primarily induced by purine dysregulation. Current therapies face limitations due to systemic side effects and inadequate efficacy, necessitating novel treatment strategies.

**Methods:**

In this study, a biomimetic nanodrug, SZF@PDA‑RM, was synthesized by hybridizing the traditional medicine Shizhifang (SZF) with polydopamine (PDA), followed by erythrocyte membrane coating. Its physicochemical properties and reactive oxygen species (ROS)-responsive release were characterized. *In vitro*, a hyperuricemia cell model using uric acid (UA)-stimulated renal tubular cells (NRK‑52E) assessed cellular uptake, biosafety, ROS scavenging, and mitochondrial protection. Molecular mechanisms were probed via immunofluorescence, western blot, and inhibitor studies. In vivo efficacy and safety were evaluated in a hyperuricemic mouse model by measuring serum/urinary biomarkers, renal histopathology, and tissue ROS.

**Results:**

The synthesized SZF@PDA‑RM exhibited a spherical morphology, demonstrating good stability and significant ROS-responsive drug release properties. The erythrocyte membrane coating effectively prolonged its systemic circulation. In the cellular model, SZF@PDA‑RM efficiently reduced UA-induced intracellular and mitochondrial ROS levels, restored mitochondrial membrane potential, mitigated mtDNA damage, and inhibited the activation of the NLRP3 inflammasome and the expression of downstream cytokines. Mechanistically, the nanoformulation negatively regulated mitochondrial ROS generation by promoting the interaction between SHP2 and ANT1, an effect that was reversed by SHP2 inhibitors. In the animal model, treatment with SZF@PDA‑RM significantly lowered serum uric acid, creatinine, and multiple urinary renal injury biomarkers in hyperuricemic mice. It also alleviated renal inflammatory infiltration and fibrosis, cleared renal tissue ROS, and showed no systemic toxicity.

**Conclusion:**

The erythrocyte membrane-camouflaged SZF@PDA‑RM nanocomposite achieves long circulation and targeted delivery. It exerts a multi-target synergistic therapeutic effect by directly scavenging ROS, maintaining mitochondrial homeostasis via the SHP2/ANT1 pathway, and inhibiting NLRP3-mediated inflammation, thereby effectively improving uric acid metabolism and alleviating renal injury.

## Introduction

Uric acid is one of the metabolic products in the human body, mainly produced by dietary intake and the breakdown of purine compounds through liver metabolism, and excreted through the kidneys and digestive tract (1–3). Under normal circumstances, the production and excretion of uric acid in the body maintain a balanced state. When purine metabolism is impaired, hyperuricemia occurs. According to statistics, the global incidence rate of hyperuricemia in 2020 was 659.3/100,000, and the total number of patients was 55.86 million. It is projected that the worldwide prevalence of hyperuricemia will affect 95.8 million people by 2050 (4, 5). Hyperuricemia can cause a series of complications, including gout, uric acid nephropathy, chronic urate nephropathy, cardiovascular disease, and neurological disorders (6–9). Beyond dietary modifications, including low-purine intake, hyperuricemia is routinely treated with xanthine oxidase inhibitors such as allopurinol and febuxostat to reduce uric acid production. However, these therapies may cause side effects, such as skin allergic reactions induced by allopurinol and an increased risk of cardiovascular events associated with febuxostat. Additionally, uricosuric drugs such as benzbromarone, although effective in certain cases, may lead to liver dysfunction. Therefore, it is crucial to identify new therapeutic strategies for hyperuricemia that reduce the occurrence of adverse effects.

Oxidative stress is defined as an imbalance between the body’s production of reactive oxygen species (ROS) and its ability to neutralize them, leading to a harmful accumulation of ROS that can damage cells (10, 11). In hyperuricemia, the accumulation of urate salts can induce mitochondrial dysfunction and increase ROS production in renal tubular epithelial cells, thereby triggering oxidative stress and inflammatory responses (12–14). In addition, oxidative stress may enhance purine metabolism, further increasing uric acid production and exacerbating hyperuricemia. Numerous studies indicate that hyperuricemia can induce kidney damage through various inflammatory signaling pathways, such as activation of the PI3K/AKT/NF-κB signaling pathway, which promotes inflammatory responses and cellular injury (15–17). Therefore, elucidating the mechanisms underlying oxidative stress and inflammation will facilitate the development of novel therapeutic strategies for hyperuricemia (18–20). The negative regulatory effects of the SHP2/ANT1 signaling pathway can reduce uric acid–induced mitochondrial ROS production in renal tubular epithelial cells, inhibit activation of the NLRP3 inflammasome, and maintain mitochondrial homeostasis, thereby helping to reduce oxidative stress and improve uric acid metabolism and renal function (21, 22). Thus, regulation of the SHP2/ANT1 signaling pathway may provide a new strategy for the treatment of hyperuricemia and its related complications.

As a traditional Chinese medicine formula, Shizhifang (SZF) represents a promising multi-target therapeutic strategy for hyperuricemia and its associated renal injury (23). Unlike single-target drugs such as xanthine oxidase inhibitors, which primarily lower serum uric acid levels, the multi-component nature of SZF enables it to simultaneously modulate downstream inflammatory–metabolic cascades and mitochondrial dysfunction (24, 25). Experimental evidence demonstrates that SZF orchestrates a synergistic protective network (26). Specifically, SZF alleviates oxidative stress by suppressing mitochondrial ROS generation via the ROS–TXNIP axis, thereby inhibiting upstream activation of the NLRP3 inflammasome (25). Subsequently, SZF directly suppresses activation of the NLRP3/Caspase-1/GSDMD pathway, reducing the maturation of IL-1β and IL-18 and mitigating pyroptosis in renal tubular epithelial cells (27). Furthermore, studies indicate that SZF can regulate the SHP2/ANT1 signaling pathway, thereby reducing cellular damage. This integrated multi-pathway mechanism confers comprehensive cytoprotection and promotes improvement of renal function. Consequently, SZF exemplifies a polypharmacology approach that addresses the complexity of hyperuricemia-induced kidney damage more holistically than conventional single-entity drugs.

Herein, we aimed to design and synthesize a erythrocyte membrane-camouflaged nanocomposite drug, designated as SZF@PDA-RM, for the treatment of hyperuricemia. The core design rationale of this study is to integrate the active components of SZF with a ROS-responsive polymer, polydopamine (PDA), and further encapsulate the composite with a natural RM, thereby constructing a biomimetic delivery system. This design is intended to leverage the RM to prolong the systemic circulation of the nanodrug, enhance its biocompatibility, and potentially enable targeted delivery to sites with elevated oxidative stress. Based on this, we hypothesize that SZF@PDA-RM can respond to the high ROS levels in the hyperuricemic microenvironment to achieve controlled release of SZF. The released SZF is expected to negatively regulate uric acid-induced mitochondrial ROS production in renal tubular epithelial cells via the SHP2/ANT1 signaling pathway, thereby inhibiting NLRP3 inflammasome activation and maintaining mitochondrial homeostasis. To validate this hypothesis, we will employ an *in vitro* model using uric acid-stimulated renal tubular epithelial cells (NRK-52E) and establish an in vivo hyperuricemic mouse model, systematically evaluating the efficacy and mechanism of SZF@PDA-RM at molecular, cellular, and whole-animal levels. This study aims to provide a novel therapeutic strategy for hyperuricemia, featuring active targeting, ROS-responsive release, and multi-pathway synergistic effects.

## Methods

### SZF extraction

Herbal decoction: The formula was composed of 30 g Plantago asiatica, 15 g Mustard Seed, 15 g Wangbuliuxing, and 15 g Winter Sunflower Seed. All medicinal materials were purchased from the Traditional Chinese Medicine Pharmacy of Shanghai Seventh People’s Hospital. Preparation of SZF Dry Powder: The dry powder was prepared by weighing decoction pieces of Plantago asiatica (origin: Jiangxi; batch number 151225), Mustard Seed (origin: Gansu; batch number L Y1505016), WangBuliuxing (origin: Hebei; batch number 140220), and Winter Sunflower Seed (origin: Anhui; batch number 160517HY) at a ratio of 2:1:1:1. Eight times the volume of 75% ethanol was added, and the mixture was reflux-extracted twice, each time for 1 h. The extracts were combined, centrifuged, concentrated, and dried to obtain the SZF dry powder. Quality control was performed by high-performance liquid chromatography analysis of the Zhifang dry powder sample.

### Synthesis of SZF@PDA

Dopamine hydrochloride (DA·HCl; 0.5 g) and SZF (0.25 g) were dissolved in 100 mL of 50% ethanol and vigorously stirred at room temperature. After stirring for 10 min, 5.0 mL of ammonia solution was added dropwise to induce dopamine self-polymerization for 2 h. The reaction mixture was then centrifuged at 13,500 rpm for 15 min, and the supernatant was removed. The precipitate was washed three times with H_2_O and the final SZF@PDA product was stored at 4°C.

### Extraction of erythrocyte membranes

Erythrocyte membranes were centrifuged at 3,000 r/min for 5 min and washed three times with phosphate-buffered saline (PBS). The cells were then dispersed in PBS (pH 7.4) containing 0.1% protease inhibitor. An ultrasonic cell disruptor was used to lyse the cells. Subsequently, the mixture was centrifuged at 3,200 g for 10 min. The obtained supernatant was centrifuged again at 80,000 g for 90 min, and the erythrocyte membranes were collected using a high-speed centrifuge.

### Synthesis and characterization of SZF@PDA-RM

SZF@PDA and erythrocyte membranes were sequentially extruded through polycarbonate membrane filters with pore sizes of 5, 3, 1, 0.4, and 0.2 μm, respectively, for five cycles to obtain SZF@PDA-RM.

After nanoparticle synthesis, dynamic light scattering (DLS) was used to measure particle size and ZETA potential. Transmission electron microscopy (TEM) was used to observe the morphological characteristics of the nanoparticles. Ultraviolet–visible (UV–vis) spectroscopy was employed to detect various components and verify successful drug loading and preparation of SZF@PDA-RM.

### Stability of nanomaterials

The materials were separately incubated in PBS, 10% fetal bovine serum (FBS), and simulated body fluid (SBF) for 0, 4, 8, 12, 24, and 48 h. Particle size and potential changes were then measured using a DLS system.

### Drug loading rate

Standard curves of SZF at different concentrations were first established using a UV spectrophotometer. The SZF content in SZF@PDA-RM was subsequently determined using a UV spectrophotometer or HPLC, and the drug loading rate was calculated according to the corresponding formula.

### ROS responsiveness and drug release

SZF@PDA and SZF@PDA-RM were treated with 1 mM H_2_O_2_ for different durations (0, 6, 12, and 24 h). The UV absorption values of treated SZF@PDA and SZF@PDA-RM were measured using a UV spectrophotometer. Samples treated for 0 and 24 h were selected for TEM preparation to observe morphological changes.

The release of SZF from SZF@PDA and SZF@PDA-RM under different conditions (PBS or 1 mM H_2_O_2_) was measured using a UV spectrophotometer at 0, 4, 8, 12, and 24 h.

### Establishment of hyperuricemia cell model

Uric acid powder (250 mg; sodium urate, purchased from Sigma, item number U2875-5G) was dissolved in 5 ml of 1 mol/L NaOH to prepare a uric acid stock solution at a concentration of 50 mg/ml. The stock solution was diluted with RPMI-1640 complete culture medium containing 10% serum to prepare uric acid culture media at concentrations of 200, 400, and 600 μg/ml. Cells were cultured in uric acid media at concentrations of 0, 200, 400, and 600 μg/ml for 24 h, and the optimal modeling concentration was selected based on the toxic effects of uric acid on rat renal tubular epithelial NRK-52E cells.

### Cell counting Kit-8 assay

NRK-52E cells were seeded at a density of 10,000 cells/well in 96-well plates and incubated with sample solutions at different concentrations for 24 h. The cytotoxicity of the materials toward NRK-52E cells was evaluated using a CCK8 assay kit.

### Live/dead cell staining

NRK-52E cells were seeded at a density of 10,000 cells/well in 96-well plates and incubated with the sample solutions for 24 h. The cells were then stained with Calcein-AM/propidium iodide (PI), and cell viability was observed under a fluorescence microscope.

### Cellular uptake

To evaluate nanoparticle uptake *in vitro*, NRK-52E cells were seeded into confocal dishes at a density of 50,000 cells/dish. After cell adhesion, the medium was replaced with fresh culture medium containing Cy7.5-labeled particles, followed by incubation for 0, 4, 8, 12, and 24 h. The cells were subsequently fixed and visualized under a fluorescence microscope for imaging.

### ROS scavenging

NRK-52E cells and hyperuricemia model cells were seeded into 24-well plates and cultured for 24 h. After adhesion, the hyperuricemia group was treated with the corresponding materials and incubated for an additional 24 h. Total intracellular ROS levels were detected using ROS fluorescent probes. MitoSOX combined with flow cytometry was used to assess mitochondrial reactive oxygen species (mtROS) production. JC-1 staining was performed to evaluate mitochondrial membrane potential, and real-time quantitative fluorescence PCR was used to assess mitochondrial DNA (mtDNA) levels.

### Determination of antioxidant capacity

To evaluate intrinsic antioxidant activity, the radical scavenging capacities of SZF and its composite SZF@PDA-RM were assessed using commercial assay kits. For the DPPH radical scavenging assay, the procedure followed the manufacturer’s instructions: samples at different concentrations were mixed with an ethanolic DPPH solution and incubated in the dark for 30 min, after which absorbance was measured at 518 nm. The ABTS^+^ radical scavenging capacity was determined by reacting the samples with a pre-generated ABTS^+^ working solution for 12 min, followed by absorbance measurement at 734 nm. All experiments were performed in triplicate, with ascorbic acid (VC) used as a positive control.

### Co-immunoprecipitation

Co-immunoprecipitation was performed to analyze the interaction between SHP2 and ANT1. NRK-52E cells from different treatment groups were lysed, and the lysates were centrifuged at 14,800 rpm for 15 min at 4°C. The supernatants were collected and incubated overnight at 4°C with gentle rotation in the presence of 1 μg of anti-SHP2 antibody. Subsequently, 5 μL of a 50% slurry of Protein A/G agarose beads was added and incubated for an additional 3 h at 4°C. The beads were washed three times with ice-cold lysis buffer. After elution by boiling in SDS–PAGE loading buffer, the immunoprecipitated complexes were analyzed by Western blot using an anti-ANT1 antibody. All experiments were conducted according to the manufacturers’ protocols, with at least three technical replicates per condition and three independent biological replicates.

### Western blot

After different treatments, cells were subjected to immunofluorescence staining and observed under confocal microscopy to assess SHP2/ANT1 colocalization in different groups.

NRK-52E hyperuricemia model cells were seeded into six-well plates and cultured. After cell adhesion, the hyperuricemia group was treated with the corresponding materials for 24 h. Western blotting and PCR were then performed to detect the protein and mRNA expression levels of Caspase-1 (p20), Caspase-1 (p10), pro-caspase-1, NLRP3, SHP2, ANT1, ASC, IL-1β, and IL-18. Hyperuricemia cells were treated with or without the SHP2 inhibitors PHPS1 (10 μM) or NSC87877 (10 μM) for 1 h, followed by incubation with SZF@PDA-RM for 24 h. The levels of IL-1β and IL-18 in the cell supernatants were measured using enzyme-linked immunosorbent assay (ELISA).

### Establishment of a hyperuricemia model in BALB/c Mice

The model group and each treatment group received intraperitoneal injections of potassium oxonate (250 mg/kg; purchased from Shandong Zhongke Taidou Chemical Co., Ltd., product number 2207-75-2) starting on the first day of the experiment, and the model was continuously established for 14 days (28, 29). Drug intervention was initiated on the first day of modeling and continued until the end of the modeling period, while the normal control group received an equal volume of physiological saline. The mouse dosage was calculated according to standard pharmacological experimental animal administration methods and was equivalent to nine times the human equivalent dose. Based on an adult standard body weight of 60 kg, the corresponding mouse dosage was 11.25 g/kg, equivalent to 11.25 mg/g (27).

### *In vivo* safety and drug circulation analysis

Healthy mice were treated with physiological saline, PDA-RM, or SZF@PDA-RM for 72 h, after which plasma samples were collected for blood biochemical analysis. Serum alanine aminotransferase (ALT), aspartate aminotransferase (AST), blood urea nitrogen (BUN), creatinine (CREA), creatine kinase (CK), and other indicators were measured. In addition, red blood cells were collected to assess the hemolytic behavior of the materials. Briefly, 200 μL of red blood cell suspension was added to PDA-RM or SZF@PDA-RM, with water used as a control. After centrifugation at 10,000 rpm for 15 min, the supernatants were collected, and absorbance was measured at 540 nm.

To compare blood circulation of SZF@PDA and SZF@PDA-RM. 6–8-week-old mice (*n*=5) were intravenously injected via the tail vein at a dose of 10 mg/kg^-1^. Blood samples were collected from the orbital venous plexus at various time points after administration to determine SZF concentrations and calculate the circulation half-life (t_1/2_=34 h).

### Evaluation of *in vivo* treatment efficacy

Twenty-four-hour urine and serum samples were collected from mice on days 0 and 14 of the experiment. Urine samples were centrifuged at 3,500 rpm for 10 min, and 200 μl of the supernatant was collected for analysis. After fasting for 12 h, pentobarbital sodium was used for intraperitoneal anesthesia. The eyeballs were removed, and blood was collected via enucleation, allowed to stand for 2 h, centrifuged at 3,500 rpm for 10 min, and the serum supernatant was collected for testing.

Routine blood and urine parameters included 24-h urinary uric acid (24-h UUA), 24-h urinary protein (UPT), urinary β2-microglobulin (β2-MG), urinary retinol-binding protein (RBP), urinary N-acetyl-β-D-glucosaminidase (NAG), urinary α 1-microglobulin (α1-MG), serum uric acid (SUA), serum creatinine (SCR), and blood urea nitrogen (BUN).

After completion of treatment, liver and kidney tissues were collected for H&E staining and Masson trichrome staining to evaluate histopathological changes.

Total ROS and mitochondrial ROS levels in renal tubular epithelial cells from each group were detected using flow cytometry. Renal tissues were also collected for immunofluorescence analysis to detect protein expression levels of Caspase-1 (p20), Caspase-1 (p10), pro-caspase-1, NLRP3, SHP2, ANT1, ASC, IL-1β, and IL-18.

## Results and discussion

### Synthesis and characterization of SZF@PDA-RM

**Figure 1:**
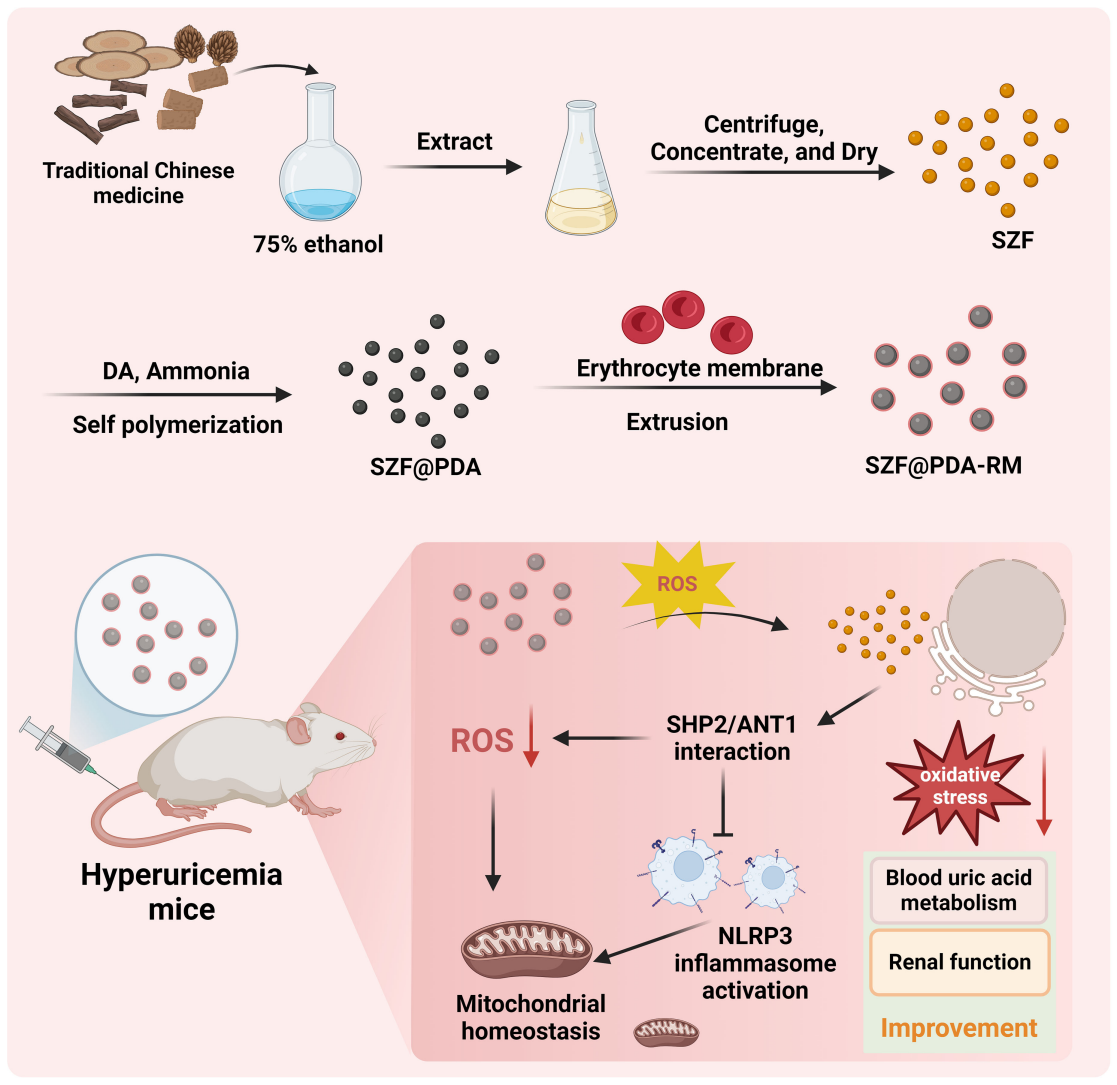
Schematic diagram illustrating SZF@PDA-RM synthesis and the mechanism by which SZF@PDA-RM inhibits activation of the NLRP3 inflammasome through the SHP2/ANT1 signaling pathway, thereby maintaining mitochondrial homeostasis, reducing oxidative stress, and improving blood uric acid metabolism and renal function.

**Figure 2:**
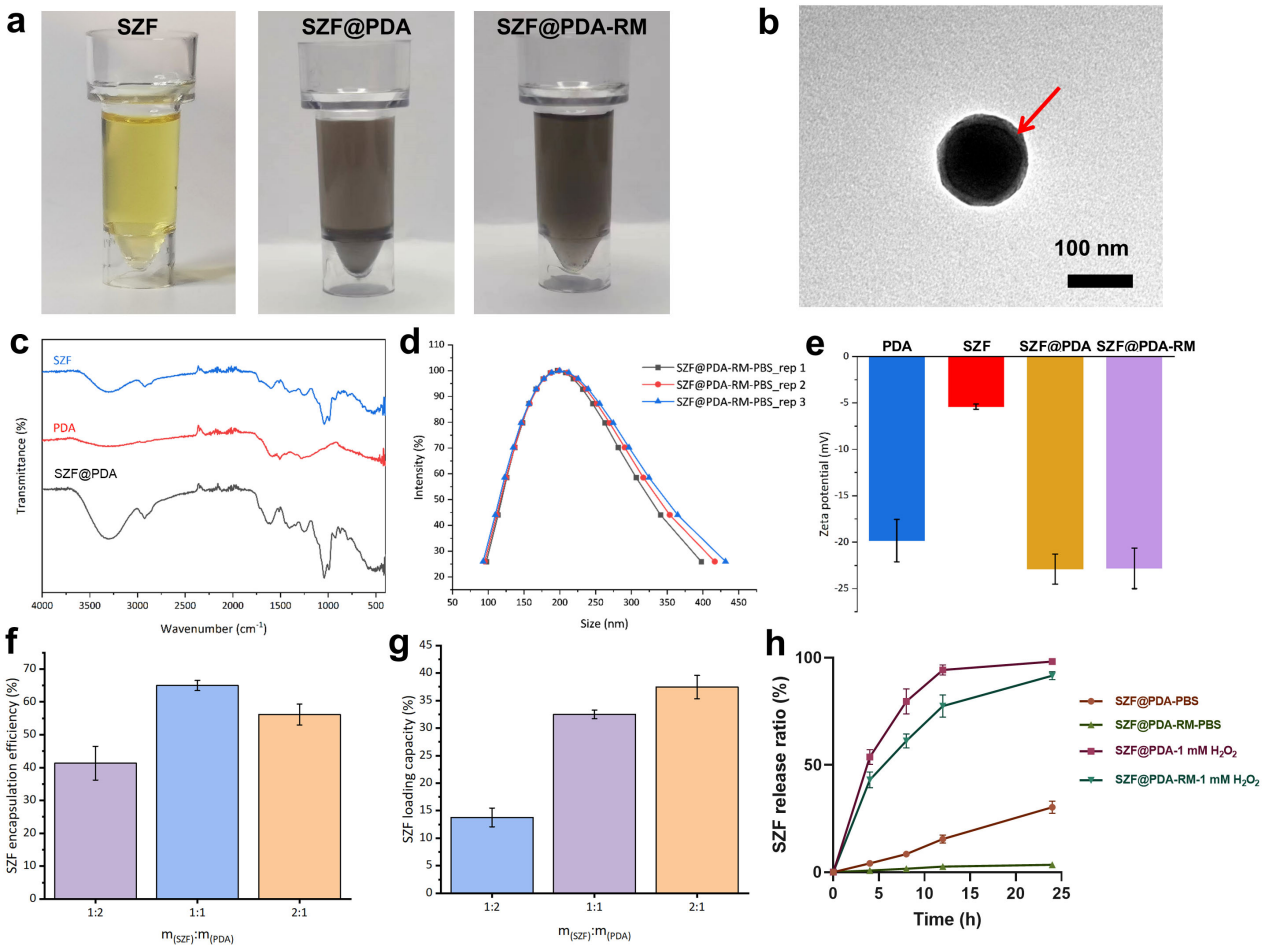
**(a)** Optical photographs of nanoparticles in ethanol solution. **(b)** Transmission electron microscopy (TEM) image of SZF@PDA-RM. **(c)** Fourier transform infrared spectroscopy spectra of SZF, PDA, and SZF@PDA. **(d)** Particle size distribution of SZF@PDA-RM. **(e)** Zeta potential of PDA, SZF, SZF@PDA, and SZF@PDA-RM. **(f, g)** Encapsulation efficiency and drug loading capacity of SZF (*n*=3). **(h)** SZF@PDA and SZF@PDA-RM H_2_O_2_ responsive release of SZF (*n*=3).

SZF was extracted from a series of Chinese herbal medicines according to previously reported work (30). Polydopamine (PDA), a polymer material with favorable biocompatibility and excellent (ROS)-scavenging ability, was hybridized with SZF through oxidative self-polymerization to form SZF@PDA. To improve blood circulation time and enhance the therapeutic efficiency of the hybrid system, SZF@PDA was further encapsulated with an erythrocyte membrane via an extrusion method (**Figure 1**). The SZF extract could be completely dissolved in a 50% ethanol solution. In contrast, SZF@PDA exhibited reduced stability in ethanol and formed precipitates after standing for a period of time, as indicated by the red arrow. However, after erythrocyte membrane encapsulation, the stability of SZF@PDA-RM was significantly improved (**Figure 2a**). Transmission electron microscopy (TEM) images after negative staining showed that SZF@PDA-RM exhibited a spherical morphology and indicated the presence of an erythrocyte membrane coating on the SZF@PDA-RM surface (**Supplementary Figure S1**, **Figure 2b**). Fourier transform infrared spectroscopy was used to verify successful encapsulation of PDA onto the SZF surface. As shown in **Figure 2c**, characteristic peaks corresponding to both PDA and SZF were present in the spectrum of SZF@PDA, confirming its successful preparation. DLS analysis further demonstrated a uniform particle size distribution centered at approximately 200 nm (**Figure 2d**). The surface charge of the systems at different modification stages was characterized by zeta potential measurements (**Figure 2e**). While native SZF exhibited a negatively charged surface, hybridization with PDA followed by erythrocyte membrane coating led to a pronounced decrease in zeta potential. Notably, erythrocyte membrane encapsulation did not markedly alter the surface potential of SZF@PDA. The consistent particle size of approximately 200 nm favors renal accumulation, whereas the erythrocyte membrane–induced shift in zeta potential may reduce opsonization and prolong the circulation half-life to 34 h. The SZF loading efficiency was measured using UV visible spectroscopy (UV-vis). Based on the SZF standard curve (**Supplementary Figure S2**), the encapsulation efficiency of SZF was calculated to be 65.0 ± 1.3%, and the drug loading capacity was 32.5 ± 0.6% (**Figures 2f, g**).

In addition, the stability of SZF@PDA-RM was evaluated in phosphate-buffered saline (PBS), fetal bovine serum (FBS), and simulated body fluid (SBF). The results showed that SZF@PDA-RM exhibited excellent stability in these environments, with minimal changes in particle size (**Supplementary Figure S3**). PDA nanoparticles function as efficient antioxidants and can be oxidized and degraded while consuming excessive ROS, thereby facilitating controlled drug release (31). Accordingly, SZF@PDA-RM is responsive to elevated ROS levels, enabling effective release of SZF under ROS stimulation. SZF@PDA and SZF@PDA-RM were incubated in PBS or hydrogen peroxide (H_2_O_2_), respectively. In PBS, SZF@PDA-RM released minimal SZF, whereas SZF@PDA released more than 20% of its loaded SZF. In contrast, the cumulative release of SZF from SZF@PDA-RM reached approximately 90% within 24 h when incubated with 1 mM H_2_O_2_ (**Figure 2h**). Importantly, compared with SZF@PDA, SZF@PDA-RM exhibited a significantly delayed initial burst release and more sustained release kinetics, with improved control over the total released amount (**Figure 2h**). These findings confirm that the erythrocyte membrane acts as an effective physical diffusion barrier, enhancing sustained-release behavior and preventing sudden burst release of the nanocomposite. TEM images further showed that after incubation with H_2_O_2_ for 24 h, the structure of SZF@PDA-RM was disrupted, indicating its ROS-responsive properties and supporting ROS-triggered SZF release (**Supplementary Figure S4**). Given that hyperuricemia is characterized by elevated ROS levels in renal tubular epithelial cells, the ROS-responsive release behavior of SZF@PDA-RM enables site-specific drug delivery, which is critical for enhancing therapeutic efficacy while reducing systemic toxicity. This was further validated in subsequent *in vitro* cellular studies.

### ROS scavenging of SZF@PDA-RM *in vitro*

As a traditional Chinese medicine compound used to treat hyperuricemia, the various active ingredients in SZF may exert synergistic effects through different mechanisms, including reducing levels of pro-inflammatory cytokines and alleviating inflammatory damage (27). Therefore, a hyperuricemia cell model using rat renal tubular epithelial NRK-52E cells was constructed to further validate the therapeutic effects of SZF@PDA-RM *in vitro*.

**Figure 3:**
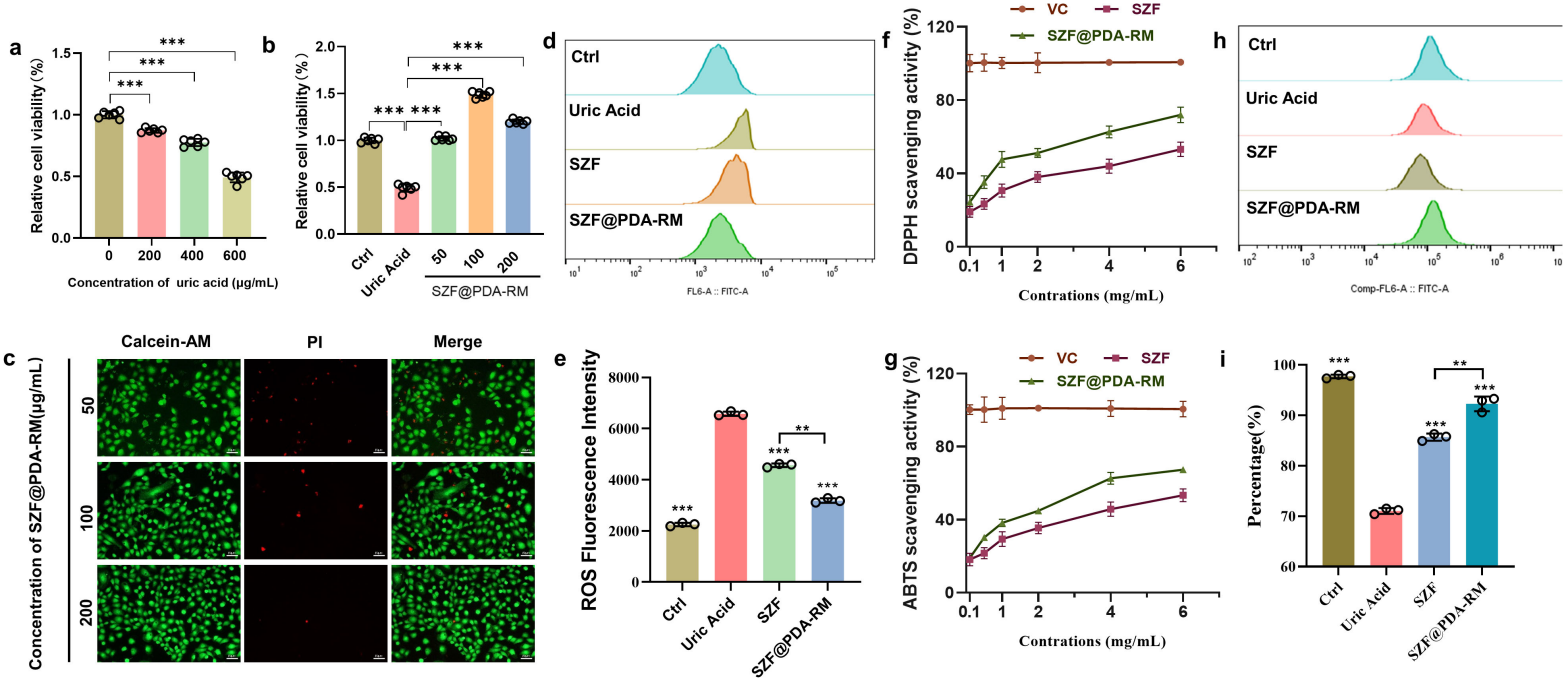
ROS scavenging of SZF@PDA-RM *in vitro*. **(a)** Cell viability of NRK-52E cells treated with different concentrations of uric acid (*n*=5). **(b)** Cell viability of NRK-52E cells in different groups, including control, uric acid, and SZF@PDA-RM (*n*=5). **(c)** Live/Dead staining of NRK-52E cells treated with different concentrations of SZF@PDA-RM. Scale bar: 50 μm. **(d, e)** Flow cytometry analysis of DCFH-DA–stained NRK-52E cells after different treatments (*n*=3). **(f)** DPPH radical scavenging activity of SZF and SZF@PDA-RM (*n*=3). **(g)** ABTS radical scavenging activity of SZF and SZF@PDA-RM (*n*=3). **(h, i)** Flow cytometry analysis of JC-1–stained NRK-52E cells after treatment (*n*=3). Data are presented as mean ± SD. Statistical significance was determined using a two-tailed Student’s t-test and one-way analysis of variance (ANOVA) with Tukey’s multiple comparisons test; **p<0.01, ***p<0.001.

Uric acid exhibits antioxidant effects at normal physiological concentrations; however, at elevated concentrations, it induces significant cytotoxicity by triggering oxidative stress and inflammatory responses (32, 33). As shown in **Figure 3a**, NRK-52E cell viability decreased markedly with increasing uric acid concentrations, with only approximately 50% cell survival observed at 600 μg/mL. Therefore, a concentration of 600 μg/mL uric acid was selected to establish the hyperuricemia cell model. Subsequently, the cytotoxicity of SZF@PDA-RM at different concentrations was evaluated using the Cell Counting Kit-8 (CCK-8) assay. No obvious cytotoxicity was observed following SZF@PDA-RM treatment (**Figure 3b**). Importantly, uric acid–induced NRK-52E cell death was significantly alleviated by SZF@PDA-RM treatment, suggesting that the SZF@PDA-RM hybrid system can protect cells from oxidative stress–induced injury. A Live/Dead cell staining assay was performed to visually confirm cell viability following SZF@PDA-RM treatment. Confocal laser scanning microscopy (CLSM) images of NRK-52E cells co-stained with Calcein-AM and PI showed minimal cell death, as evidenced by sparse red fluorescent signal appeared in the views while extensive green fluorescence following SZF@PDA-RM treatment, further demonstrating the biosafety of the SZF@PDA-RM hybrid system (**Figure 3c**). To investigate cellular uptake of SZF@PDA-RM can be engulfed by NRK-52E cells, we labeled the nanoparticles were labeled with Cy7.5 fluorescence. The results show that after co incubation with cells for 1 h, red fluorescence labeled with SZF@PDA-RM were detected within cells after 1 h of incubation. After 5 h, a substantial accumulation of nanoparticles was observed, indicating that the cells can effectively uptake of SZF@PDA-RM (**Supplementary Figure S5**).

Oxidative stress is a key contributor to cellular injury and can exacerbate hyperuricemia. Next, we employed 2’, 7’-dichlorodihydrofluorescein diacetate (DCFH-DA) staining to assess intracellular ROS levels of NRK-52E cells. As shown in **Figure 3d**, flow cytometry analysis results evidence that intracellular ROS noticeably increase in intracellular ROS following uric acid stimulation. Treatment with SZF alone partially reduced ROS production, indicating its intrinsic antioxidant potential. Notably, ROS levels in uric acid–treated NRK-52E cells were markedly reduced in the SZF@PDA-RM group, demonstrating the synergistic ROS-scavenging effects of SZF and PDA and highlighting the strong cytoprotective capacity of the hybrid system against oxidative stress–induced damage. Quantitative fluorescence analysis further confirmed the enhanced ROS scavenging efficiency of SZF@PDA-RM (**Figure 3e**). To directly evaluate free radical scavenging capacity at the chemical level, DPPH and ABTS^+^ radical scavenging assays were performed. As shown in **Figure 3f**, SZF exhibited concentration-dependent DPPH radical scavenging activity. In contrast, SZF@PDA-RM demonstrated significantly enhanced scavenging capacity at all tested concentrations. The half-maximal inhibitory concentration (IC_50_) of SZF@PDA-RM was 3.018 mg/mL, which was notably lower than that of SZF (4.179 mg/mL). Similarly, in the ABTS^+^ radical scavenging assay (**Figure 3g**), SZF@PDA-RM exhibited substantially higher scavenging efficiency than SZF alone. These chemical assay results indicate that PDA modification enhances the intrinsic radical scavenging ability of the nanocomposite and corroborates the synergistic antioxidant effects observed at the cellular level. Collectively, SZF@PDA-RM exerts a dual protective mechanism by combining strong direct radical scavenging capacity with intracellular antioxidant effects to mitigate oxidative stress–induced cellular injury.

Elevated uric acid levels can induce excessive mitochondrial ROS production in renal tubular epithelial cells, leading to mitochondrial oxidative stress, activation of inflammatory responses, and exacerbation of hyperuricemia (34). MitoSOX is a positive charge, and due to the action of mitochondrial membrane potential, the positively charged MitoSOX dye is attracted and accumulated within the mitochondria. Under normal circumstances, MitoSOX is non fluorescent. However, when it encounters superoxide anions, it forms ethidium, which binds to nucleic acids and emits fluorescence. Therefore, mitochondrial superoxide levels can be assessed based on fluorescence intensity. The results showed that uric acid stimulation significantly increased mitochondrial superoxide levels compared with the control group. SZF@PDA-RM treatment markedly reduced mitochondrial superoxide levels, demonstrating its strong mitochondrial ROS scavenging capacity (**Supplementary Figure S6**). JC-1 dye is a commonly used fluorescent probe for detecting mitochondrial membrane potential and mitochondrial protection by SZF@PDA-RM. In healthy mitochondria, JC-1 forms aggregates that emit red fluorescence, whereas mitochondrial damage leads to JC-1 monomer formation with green fluorescence emission. As shown in **Figures 3h, i**, uric acid treatment caused severe mitochondrial oxidative damage, characterized by reduced red fluorescence intensity. In contrast, SZF@PDA-RM effectively eliminated excessive ROS, restored mitochondrial membrane potential, and protected mitochondria from oxidative stress–induced damage.

Hyperuricemia is commonly associated with increased intracellular oxidative stress and excessive ROS production, which can damage mitochondria and subsequently affecting the stability and replication. Therefore, mtDNA damage was further assessed using real-time quantitative PCR (qPCR). As shown in **Supplementary Figure S7**, uric acid treatment resulted in increased mtDNA content in qPCR, indicating the oxidative stress occurred in cell mitochondria, ultimately leading to mitochondrial damage. Following SZF@PDA-RM treatment, mtDNA levels were restored toward those of the control group. Collectively, these findings demonstrate that SZF@PDA-RM effectively alleviates uric acid–induced cellular damage and supports its potential as a therapeutic strategy for hyperuricemia.

### The mechanism of SZF@PDA-RM in hyperuricemia treatment

**Figure 4:**
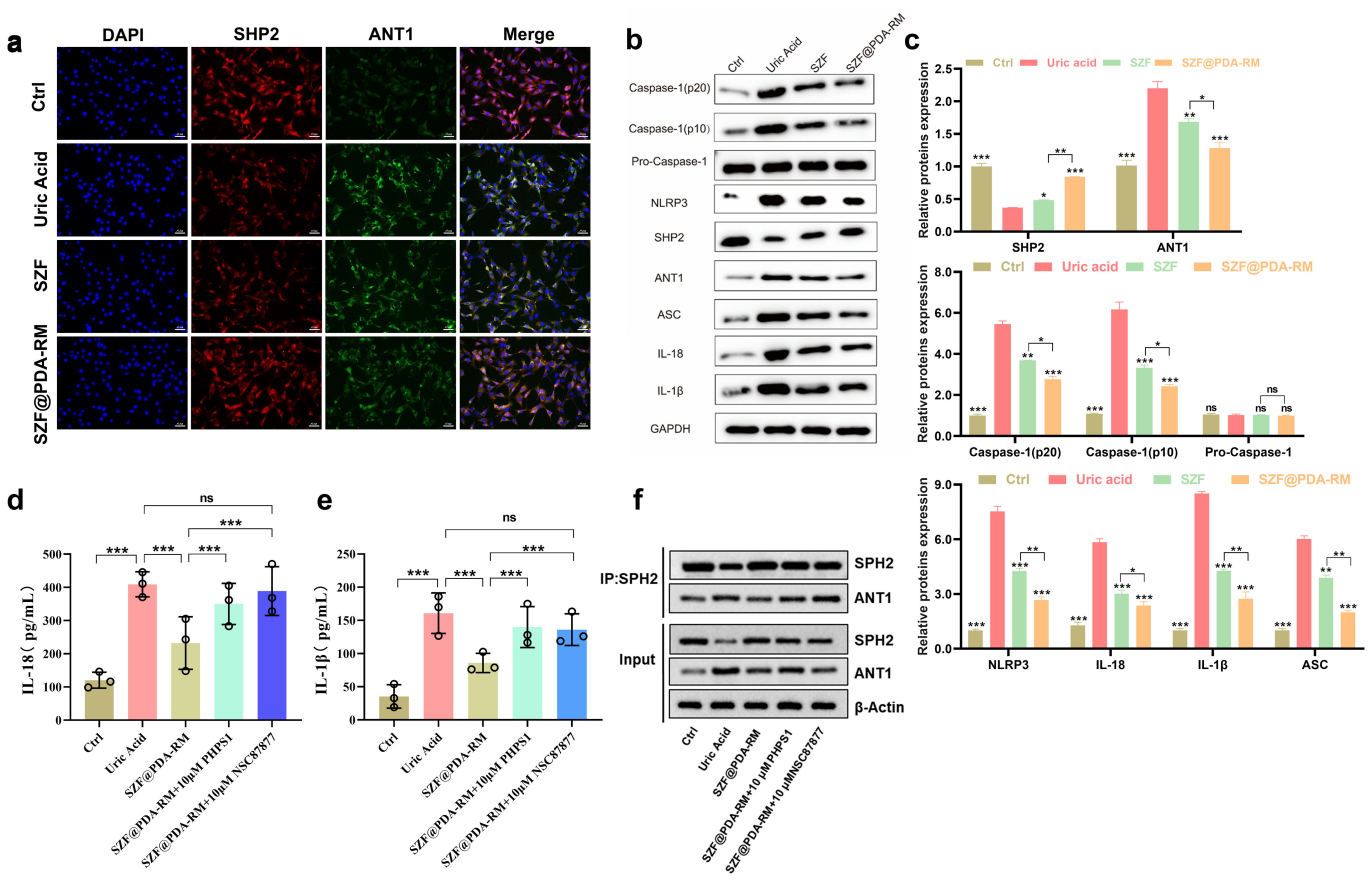
Mechanism of SZF@PDA-RM in hyperuricemia treatment. **(a)** Confocal microscopy images of SHP2/ANT1 colocalization determined by immunofluorescence staining. Scale bar: 50 μm. **(b)** Western blot (WB) analysis of protein expression in NRK-52E cells after different treatments. **(c)** Quantification of protein band intensities using ImageJ (*n*=3). **(d)** Expression levels of IL-1β in different groups determined by ELISA (*n*=3). **(e)** Expression levels of IL-18 in different groups determined by ELISA (*n*=3). **(f)** Co-immunoprecipitation (Co-IP) analysis evaluating the effect of SHP2 inhibition on SHP2–ANT1 complex formation (*n*=3). Data are presented as mean ± SD. Statistical significance was calculated using one-way ANOVA with Tukey’s multiple comparisons test; *p<0.05, **p<0.01, ***p<0.001; ns, not significant, compared with the uric acid group.

SHP2 (Src homology 2 domain–containing phosphatase 2) and ANT1 (adenine nucleotide translocase 1) play important roles in cellular signaling. SHP2 regulates mitochondrial homeostasis and inhibits activation of the NLRP3 inflammasome by interacting with ANT1, thereby alleviating hyperuricemia-related inflammatory responses and cellular stress (35). Therefore, we next explored whether SZF@PDA-RM exerts its therapeutic effects through the SHP2/ANT1 signaling pathway. Immunofluorescence staining was used to detect the colocalization of SHP2 and ANT1 in different treatment groups. As shown in **Figure 4a**, in the SZF@PDA-RM treatment group, SHP2 and ANT1 exhibited pronounced intracellular colocalization, indicating a strengthened interaction between SHP2 and ANT1.

Western blot (WB) analysis was performed to detect intracellular protein expression levels (**Figures 4b, c**). Caspase-1 is a key enzyme involved in inflammasome activation, and its active forms, Caspase-1 (p20) and Caspase-1 (p10), are closely associated with inflammatory responses. In the uric acid group, the expression levels of Caspase-1 (p20) and Caspase-1 (p10) were increased, indicating that elevated uric acid induces inflammasome activation and inflammation. In the SZF and SZF@PDA-RM groups, the levels of Caspase-1 (p20) and Caspase-1 (p10) were reduced, suggesting that the treatments effectively inhibited Caspase-1 activation and attenuated inflammatory responses. The expression level of Pro-Caspase-1 remained relatively stable across groups, indicating that drug treatment did not significantly affect total Caspase-1 expression but rather influenced its activation process. NLRP3 and ASC are essential components of the inflammasome complex. In the uric acid group, the expression of NLRP3 increased, while in the SZF@PDA-RM group, the expression of NLRP3 is significantly reduced, implying that it may alleviate the inflammatory response by inhibiting the expression, suggesting suppression of inflammasome assembly. In addition, the expression levels of the pro-inflammatory cytokines IL-18 and IL-1 β increase, while SZF and SZF@PDA-RM treatment, especially SZF@PDA-RM treatment decreased their expression, further indicating that SZF@PDA-RM effectively inhibits uric acid–induced inflammatory responses.

To further demonstrate that SZF@PDA-RM negatively regulates uric acid–induced mitochondrial ROS production and inflammation through the SHP2/ANT1 signaling pathway, we compared IL-1 β and IL-18 levels in the presence and absence of SHP2 inhibitors (PHPS1 and NSC87877). Compared with the uric acid group, the levels of IL-1 β and IL-18 in the SZF@PDA-RM treatment significantly reduced, indicating that SZF@PDA-RM can effectively inhibit uric acid induced inflammatory effect (**Figures 4d,e**). However, compared with the SZF@PDA-RM treatment group alone, the intracellular levels of IL-1 β and IL-18 did not significantly decrease after the addition of inhibitors, and may even reversed, indicating that SHP2 inhibition counteracts the anti-inflammatory effect of SZF@PDA-RM. Due to the use of SHP2 inhibitors, inflammation levels cannot be effectively restored, which further confirms that disruption of SHP2 function impairs this protective effect. To directly assess whether SZF@PDA-RM modulates upstream signaling interactions, co-immunoprecipitation (Co-IP) was performed to evaluate the effect of SHP2 inhibition on the SHP2–ANT1 interaction. As shown in **Figure 4f**, pretreatment with PHPS1 or NSC87877 markedly attenuated the SZF@PDA-RM–induced enhancement of SHP2–ANT1 binding. This finding indicates that SZF@PDA-RM promotes the SHP2–ANT1 interaction in an SHP2 activity–dependent manner. Collectively, these data support the conclusion that SZF@PDA-RM suppresses uric acid–induced mitochondrial ROS production and downstream inflammatory responses primarily through activation of the SHP2/ANT1 signaling axis.

### The hyperuricemia treatment effect of SZF@PDA-RM *in vivo*

**Figure 5:**
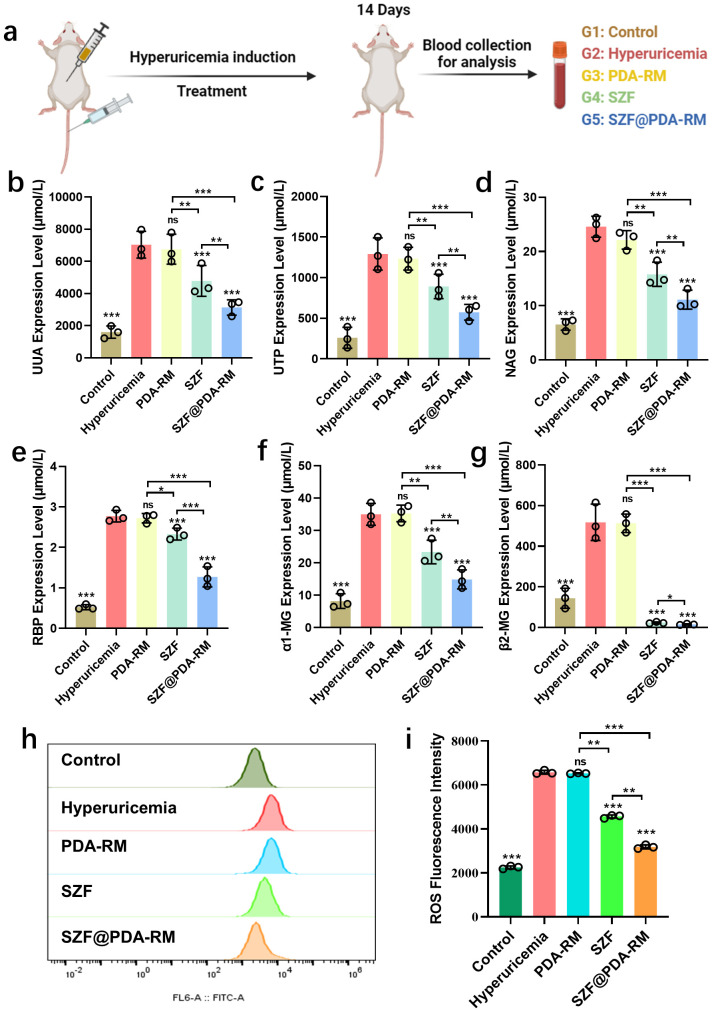
Therapeutic effect of SZF@PDA-RM on hyperuricemia *in vivo*. **(a)** Schematic illustration of SZF@PDA-RM–mediated therapy in hyperuricemia mice. **(b–g)** Blood and urine biochemical parameters of mice after different treatments (*n*=3). **(h, i)** Flow cytometry analysis of total ROS levels and corresponding fluorescence quantification in renal tubular epithelial cells from mice treated with different regimens (*n*=3). Data are presented as mean ± SD. Statistical significance was determined using one-way ANOVA with Tukey’s multiple comparisons test; *p<0.05, **p<0.01, ***p<0.001; ns, not significant, compared with the uric acid group.

After demonstrating that SZF@PDA-RM negatively regulates uric acid–induced inflammatory responses through the SHP2/ANT1 signaling pathway, thereby reducing oxidative stress and improving uric acid metabolism and renal function, we constructed a hyperuricemia model in BALB/c mice to further evaluate the therapeutic effects of SZF@PDA-RM *in vivo* (**Figure 5a**). Prior to efficacy evaluation, an *in vivo* safety assessment of PDA-RM and SZF@PDA-RM was performed. After 72 h of treatment in healthy mice, plasma samples were collected for blood biochemical analysis. The results of serum ALT, AST, BUN, CREA, CK, and other indicators showed that SZF@PDA-RM did not induce systemic toxicity (**Supplementary Figure S8**).

SZF@PDA-RM was administered to hyperuricemia mice via tail vein injection, and 24-h urine and serum samples were collected on days 0 and 14 for routine blood and urine analyses. Compared with healthy mice, hyperuricemia model mice exhibited significantly elevated levels of 24-UUA, UPT, urinary NAG, RBP, α1-MG, β2-MG, BUN, SCR, and SUA. Following SZF@PDA-RM treatment, the levels of these indicators were significantly reduced, indicating a pronounced therapeutic effect of SZF@PDA-RM on hyperuricemia and associated renal dysfunction (**Figures 5b–g**, **Supplementary Figure S9**). Notably, the marked reductions in serum and urinary uric acid levels suggest that SZF@PDA-RM modulates uric acid homeostasis primarily by promoting uric acid excretion rather than inhibiting its production (**Figure 5b**, **Supplementary Figure S9b**).

H&E staining and Masson trichrome staining were performed to assess renal pathological changes (**Supplementary Figure S10**). H&E staining revealed that hyperuricemia induced renal tubular injury, inflammatory cell infiltration, cellular necrosis, and apoptosis. In contrast, SZF@PDA-RM treatment markedly alleviated these pathological changes, indicating effective renal protection. Masson trichrome staining showed extensive blue-stained collagen deposition in the hyperuricemia group, reflecting increased fibrosis, whereas SZF@PDA-RM treatment substantially reduced collagen accumulation, indicating attenuation of renal fibrosis. Furthermore, total intracellular ROS and mitochondrial ROS release of renal tubular epithelial cells in each group are detected by flow cytometry. The results indicate that SZF@PDA-RM can effectively eliminate total ROS and mitochondrial ROS in renal tubular epithelial cells, thereby contributing to the therapeutic effect against hyperuricemia (**Figures 5h, i**). Kidney tissues were subsequently collected for immunofluorescence analysis of inflammation-related protein expression. The results showed that SZF@PDA-RM treatment significantly reduced the expression levels of inflammatory markers (**Supplementary Figure S11**). Collectively, these findings demonstrate that SZF@PDA-RM exhibits robust ROS-scavenging and anti-inflammatory effects *in vivo*, effectively ameliorating hyperuricemia progression and renal injury.

## Conclusion

In this study, we successfully developed a novel nanocomposite drug, SZF@PDA-RM, which synergistically improves uric acid metabolism and renal function through multi-target mechanisms. Specifically, SZF modulates the SHP2/ANT1 signaling pathway to suppress mitochondrial ROS production at its source, PDA serves as an immediate ROS scavenger to mitigate acute oxidative stress, and the erythrocyte membrane coating prolongs systemic circulation and reduces immunogenicity, thereby enhancing delivery efficiency and therapeutic efficacy. Together, these integrated functions—including ROS clearance, inflammation inhibition, and maintenance of mitochondrial homeostasis—provide a promising and comprehensive therapeutic strategy for the treatment of hyperuricemia.

## Data Availability

The original contributions presented in the study are included in the article/**Supplementary Material**. Further inquiries can be directed to the corresponding author.
