# Supplementary material for: Erythrocyte membrane-encapsulated SZF nanocomposites for hyperuricemia therapy
Source: Front Immunol. 2026 Jan 21;16:1695253. doi: 10.3389/fimmu.2025.1695253 (PMC12867864; doi:10.3389/fimmu.2025.1695253)
Supplement: Supplementary file 1 [file Table1.docx]

Supporting Information


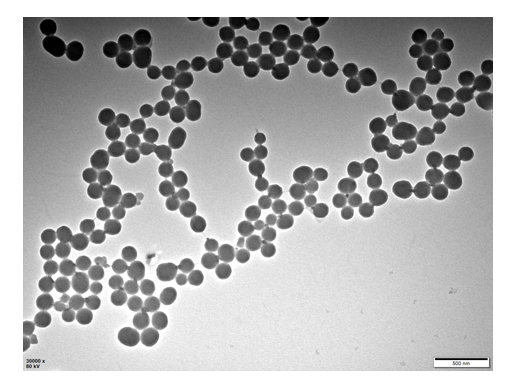


**Figure S1**. SEM images of SZF@PDA-RM.


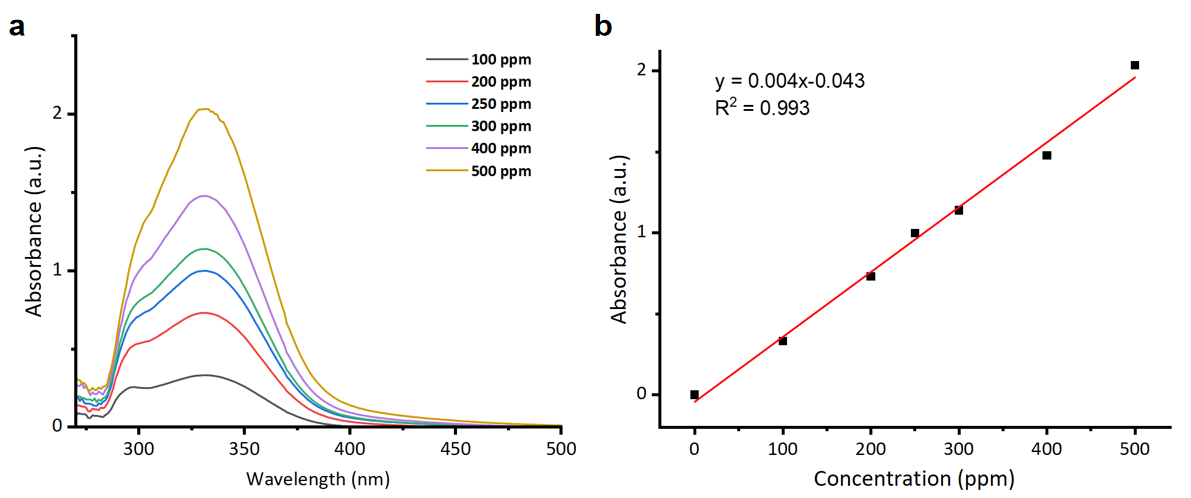


**Figure S2**. (a) UV-vis absorption spectrum of SZF in different concentration. (b) The standard curve obtained from SZF concentration and corresponding UV-vis absorbance at 332 nm.


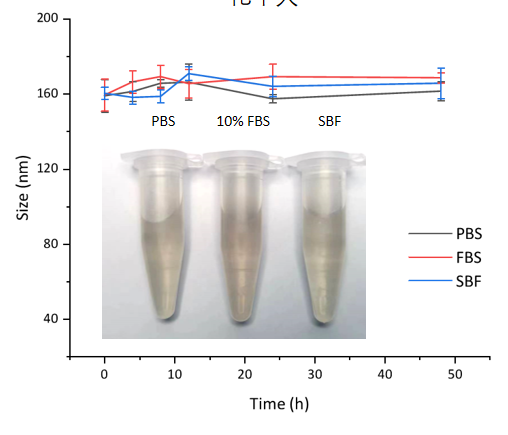


**Figure S3**. The stability of SZF@PDA-RM in PBS, 10% FBS, and SBF (n=3).


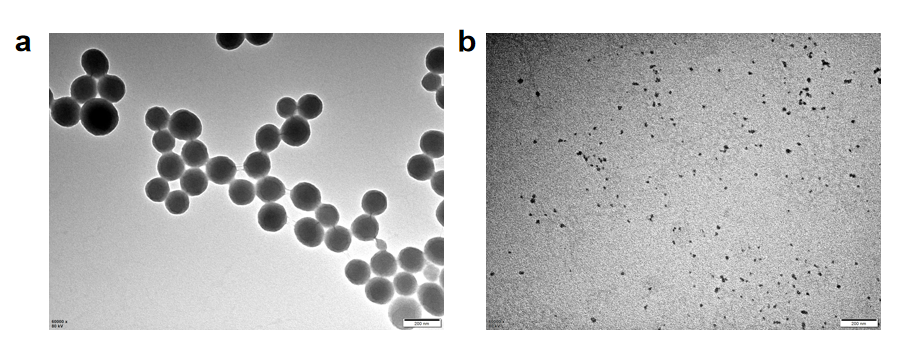


**Figure S4**. TEM image of SZF@PDA-RM incubated with 1mM H_2_O_2_ for 0 h. (b) TEM image of SZF@PDA-RM incubated with 1mM H_2_O_2_ for 24 h.


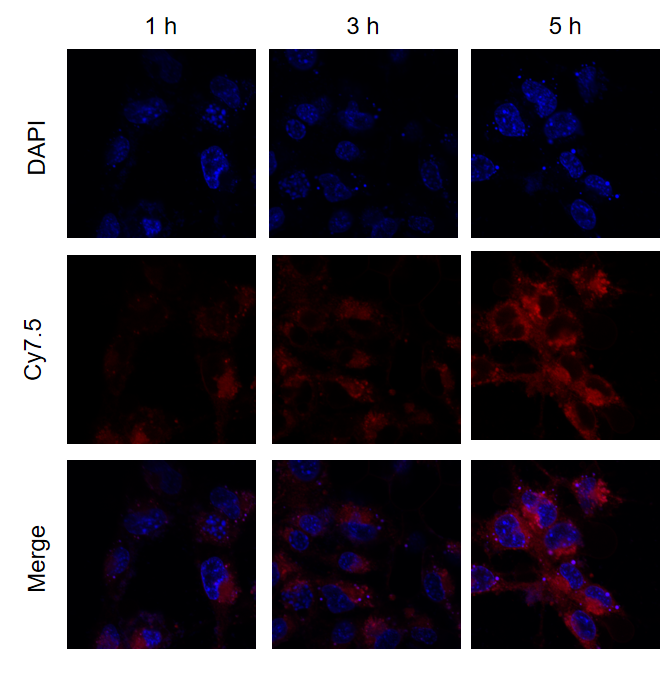


**Figure S5**. CLSM of NRK-52E cell uptake of cy7.5 labeled SZF@PDA-RM.


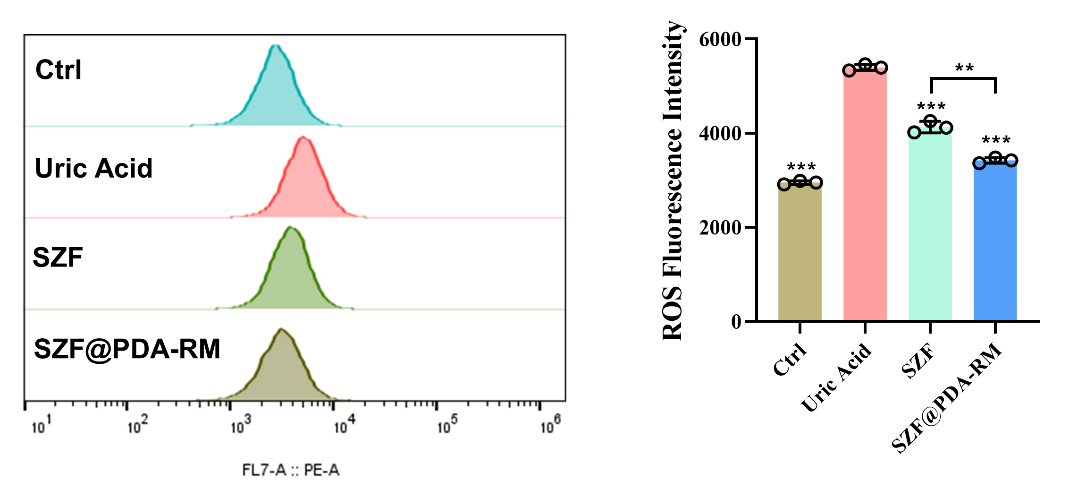


**Figure S6**. Flow cytometry analysis and corresponding fluorescence quantitative analysis of HNRK-52E cells stained with MitoSOX after different treatments (*n* = 3). The data are presented as mean value ± SD. Statistical significance was calculated using one-way analysis of variance (ANOVA) with Tukey multiple comparison, ***p* <0.01, ****p* <0.001, compared with Uric Acid Group.


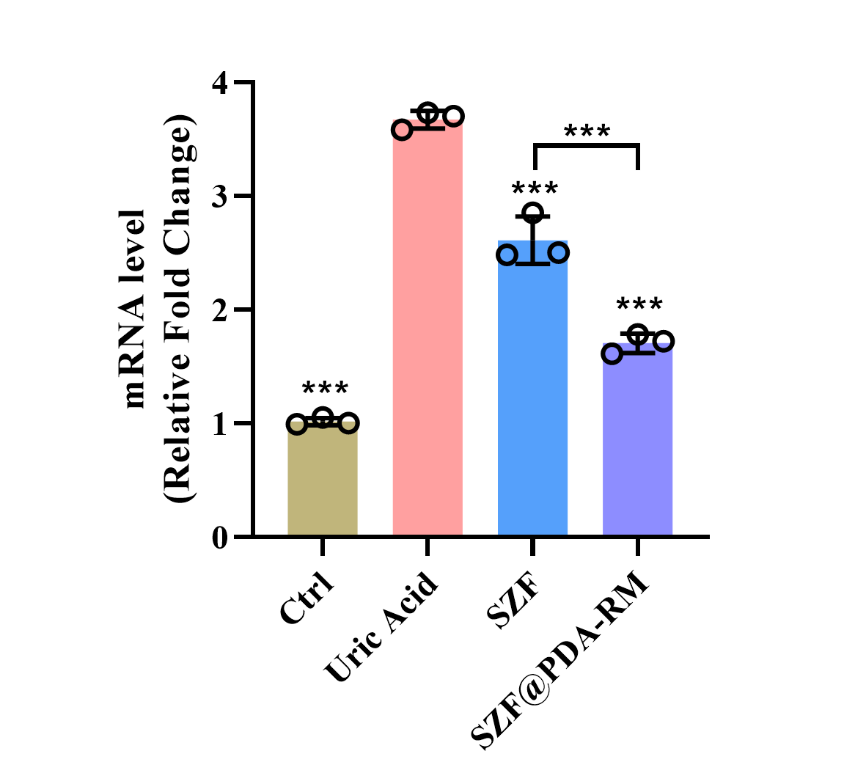


**Figure S7**. The levels of mitochondrial DNA (mtDNA) in cells after different treatments using qPCR (n=3). The data are presented as mean value ± SD. Statistical significance was calculated using one-way analysis of variance (ANOVA) with Tukey multiple comparison, ****p* <0.001, compared with Uric Acid Group.


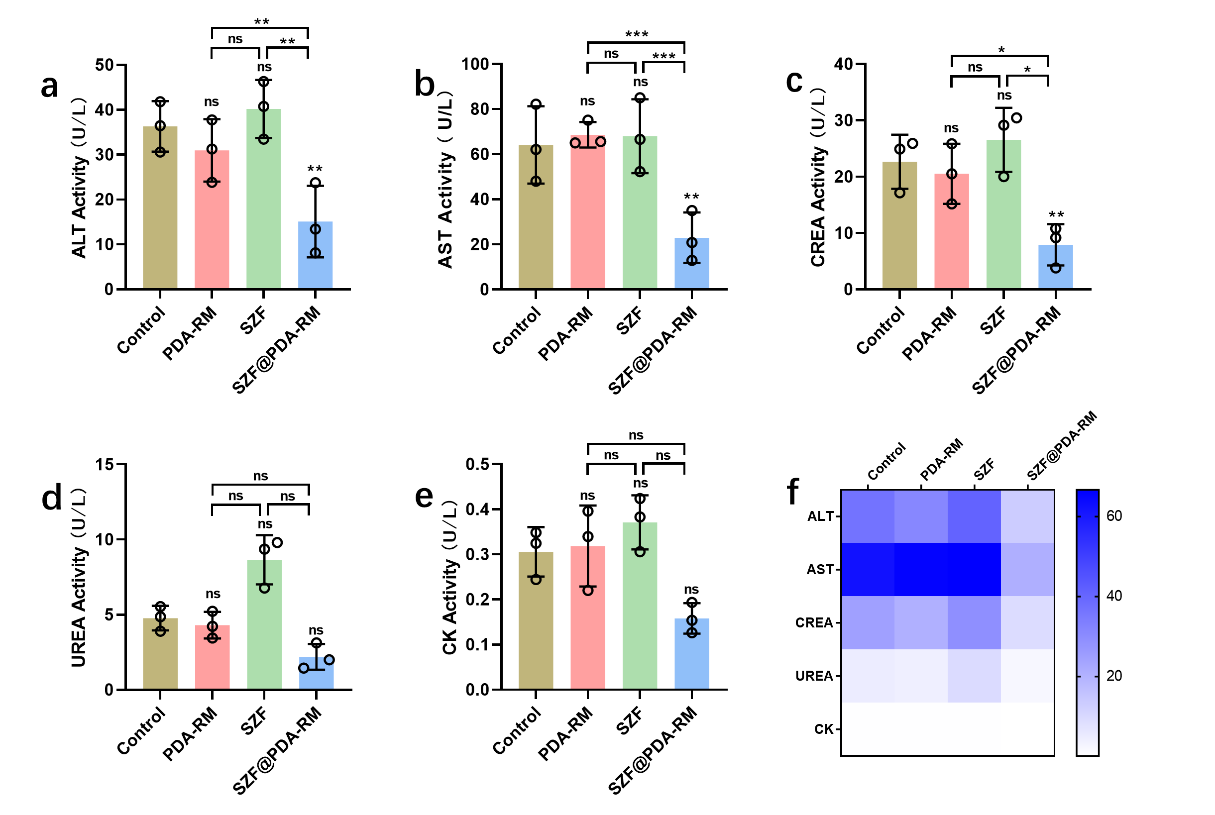


**Figure S8**. Blood biochemical analysis results of mice after different treatments, including control, PDA-RM, SZF, and SZF@PDA-RM (*n* = 3). The data are presented as mean value ± SD. Statistical significance was calculated using one-way analysis of variance (ANOVA) with Tukey multiple comparison, **p* <0.05, ***p* <0.01, ****p* <0.001, ns, no significant, compared with Control Group.


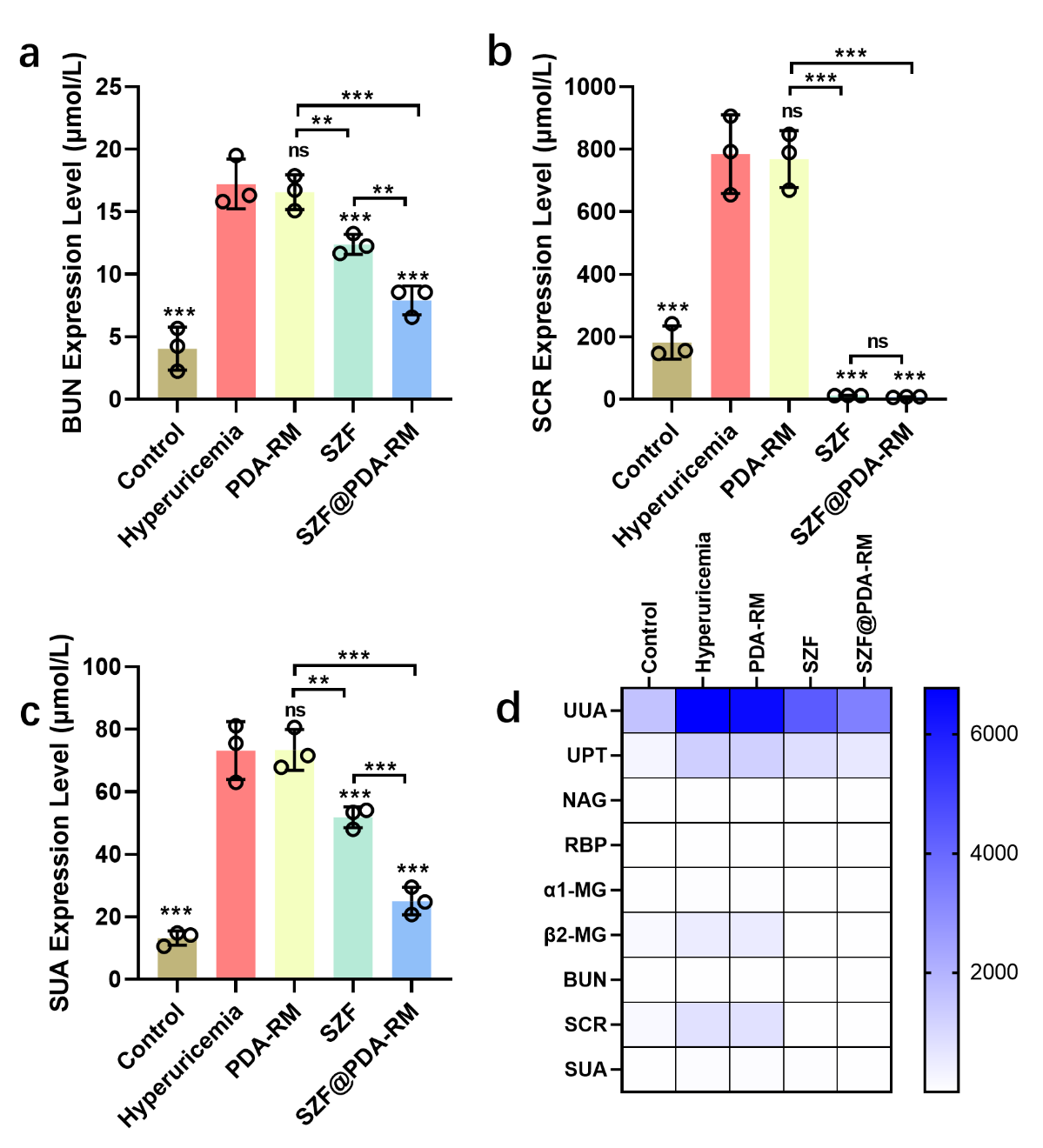


**Figure S9**. Blood and urine routine test results of mice after different treatments (*n* = 3). Statistical significance was calculated using one-way analysis of variance (ANOVA) with Tukey multiple comparison, **p* <0.05, ***p* <0.01, ****p* <0.001, ns, no significant, compared with Uric Acid Group.


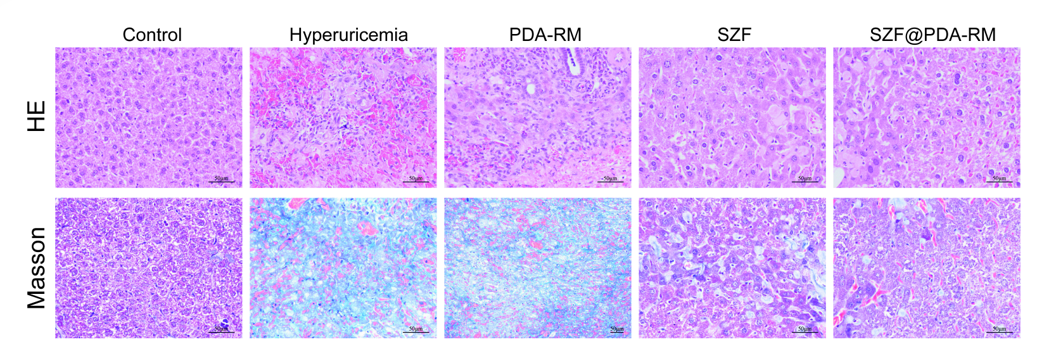
**Figure S10**. H&E staining and Masson trichrome staining of kidney tissues for evaluating the kidney injury in different groups.


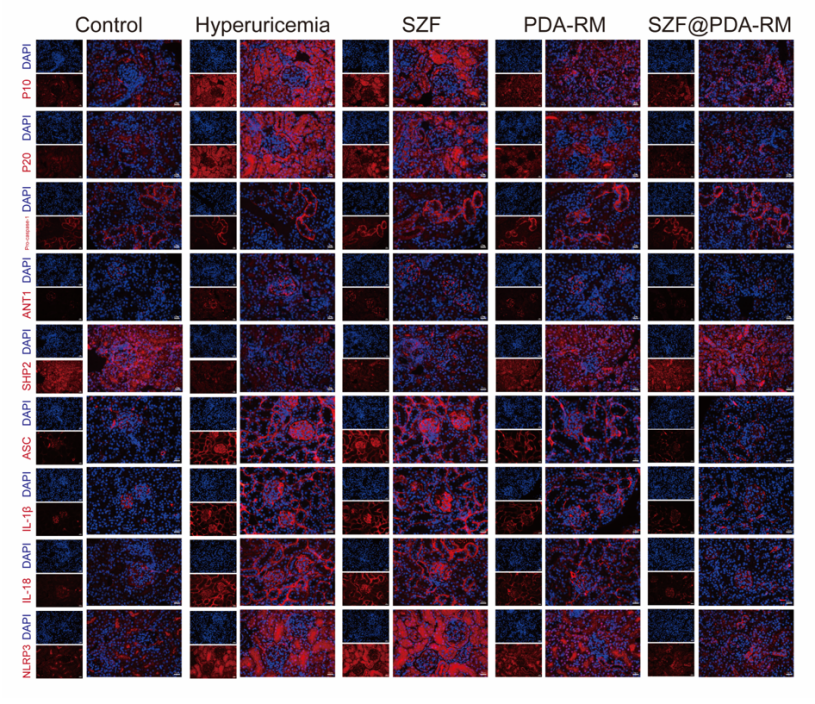


**Figure S11**. Immunofluorescence detection results of mouse kidney tissue after different treatments.
